# Glass shape influences drinking behaviours in three laboratory experiments

**DOI:** 10.1038/s41598-020-70278-6

**Published:** 2020-08-07

**Authors:** Tess Langfield, Rachel Pechey, Philippe T. Gilchrist, Mark Pilling, Theresa M. Marteau

**Affiliations:** 1 Behaviour and Health Research Unit, Department of Public Health and Primary Care, University of Cambridge, Cambridge, CB2 0SR UK; 2 Centre for Emotional Health, Department of Psychology, Macquarie University, Macquarie Park, NSW 2109 Australia; 3 MRC/BHF Cardiovascular Epidemiology Unit, Department of Public Health and Primary Care, University of Cambridge, Cambridge, CB1 8RN UK

**Keywords:** Psychology, Human behaviour

## Abstract

Reducing consumption of drinks which contain high levels of sugar and/or alcohol may improve population health. There is increasing interest in health behaviour change approaches which work by changing cues in physical environments (“nudges”). Glassware represents a modifiable cue in the drinking environment that may influence how much we drink. Here, we report three laboratory experiments measuring consumption of soft drinks served in different glasses (straight-sided vs. outward-sloped), using distinct paradigms to measure drinking. In Study 1 (N = 200), though total drinking time was equivalent, participants consumed a soft drink with a more ‘decelerated’ trajectory from outward-sloped tumblers, characterised by a greater amount consumed in the first half of the drinking episode. In Study 2 (N = 72), during a bogus taste test, participants consumed less from straight-sided wine flutes than outward-sloped martini coupes. In Study 3 (N = 40), using facial electromyography to explore a potential mechanism for decreased consumption, straight-sided glasses elicited more ‘pursed’ lip embouchures, which may partly explain reduced consumption from these glasses. Using a combination of methods, including objective measures of volume drunk and physiological measures, these findings suggest that switching to straight-sided glasses may be one intervention contributing to the many needed to reduce consumption of health-harming drinks.

## Introduction

Overconsumption of drinks containing excess sugars and alcohol is a major threat to population health globally^1-4^. Sugary drink consumption, in particular, is linked with a number of health conditions, including Type 2 diabetes, cardiovascular disease, and others^5,6^. Developing novel and effective interventions to change drinking behaviour is thus an important goal of research and policy. There is increasing interest in approaches that work by changing cues in physical environments—known also as “nudging”^7-10^. Broadly speaking, these interventions are thought to engage automatic (rather than reflective) processes, requiring relatively less active engagement or high-level cognitive processing to elicit a change in behaviour than other types of behaviour change techniques^11^. One aspect of the drinking environment that has the potential to influence drinking behaviour—possibly outside of awareness—is the glassware in which drinks are served.

There is a growing evidence base for the effect of glass size and shape on drinking behaviours. Wine glass size has increased over the past 300 years—in particular in the last 30 years^12^—with some evidence that the use of larger wine glasses increases wine consumed^13-15^. The shape of a glass—in particular, whether it is outward-sloped or straight-sided—may also influence consumption. Two studies have explored the impact of glass shape on the total time spent drinking. One study found slower consumption from straight-sided glasses for beer served in beer glasses, though no evidence that soft drink consumption differed^16^. These authors argued that the effects may be stronger for alcohol vs. soft drinks generally. The second study did find slower consumption from straight-sided glasses for a soft drink served in tumblers^17^. Further research is required to determine whether soft drink consumption differs, whether straight-sided glasses also reduce the amount consumed (of key interest for health behaviour change interventions), and what the underlying mechanisms might be.

Characterising ‘mechanisms of action’ is a key goal of behaviour change intervention research^18^. Gaining the much needed insight into the mechanisms of drinking behaviour will foster the development of more targeted, effective, and possibly less expensive approaches to reducing intake of unhealthy drinks (including soft drinks). Several mechanisms might contribute to the effects of glassware design on consumption. One concerns biases in visual perception. Specifically, when estimating the volume remaining in a drink, people may use height as a cue to volume^19^. For example, the true midpoint of drinks presented in outward-sloped glasses is underestimated, as compared to straight-sided ones^16,17,20^. This midpoint bias reflects an inability to make accurate visual judgments about the volume remaining in the glass, which might in turn influence drinking speed and amount consumed.

Another possible mechanism concerns the physical characteristics of the container such that glasses of certain shapes cue or ‘afford’ larger or smaller sip sizes and/or other micro-drinking behaviours affecting consumption. For example, there is some evidence that glasses of different shapes lead to different drinking trajectories, with more decelerated drinking found from short, wide glasses than from tall, narrow ones^21^. Decelerated drinking is characterised by a greater amount being consumed in the first half of the drinking episode. Characterising these trajectories may help understand the mechanisms that drive the effects of glass shape on consumption.

One way that affordance of glass shape on drinking may operate is via the position of the lips—‘embouchures’—and in particular, the extent to which they are pursed during sipping. The orbicularis oris muscle is responsible for compressing the lips and protruding them forward into a pucker^22^. Activity in these muscles has been found to distinguish subtle differences in embouchures of musicians playing brass and wind instruments^23-25^. To our knowledge, only one study has investigated the impact of the receptacle from which a drink is consumed on activity in the orbicularis oris muscle^26^. These researchers measured lip muscle activity using facial electromyography and found higher levels of muscle activity—indicative of a more pursed embouchure—when sips were taken through a straw, as compared to those taken from a cup or a spoon. However, there is an absence of evidence characterising the embouchures associated with drinking from differently-shaped glasses and the relationship between embouchure and sip size.

### The present research

The present set of studies aimed to estimate the effects of glass shape—straight-sided vs. outward-sloped—on several drinking behaviours during consumption of soft drinks, measured in laboratory studies using distinct paradigms to measure drinking, including direct observation and physiological measures.

**Figure 1:**
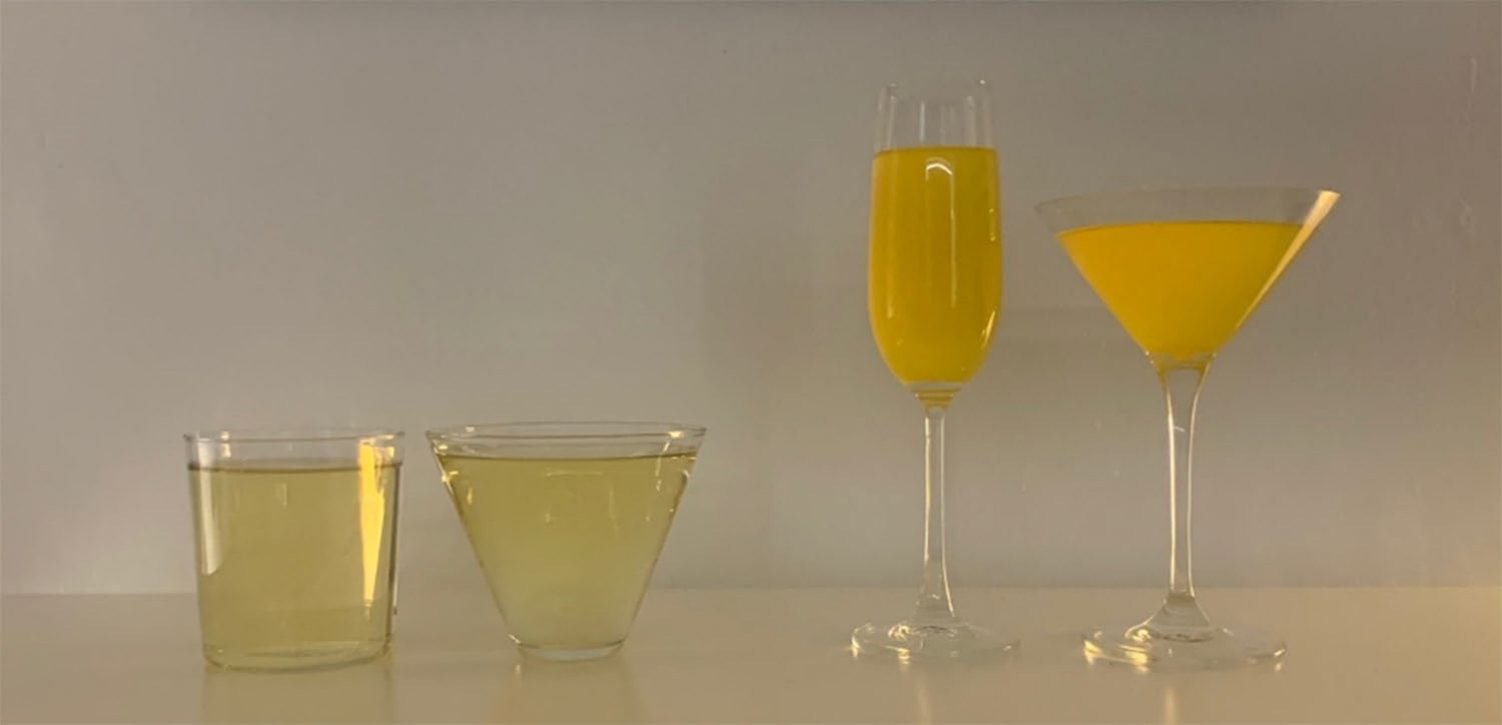
From left to right: glasses used in Study 1—straight-sided and outward-sloped tumblers containing 330 ml carbonated apple drink, and Studies 2 and 3—straight-sided wine flutes and outward-sloped martini coupes containing 165 ml non-carbonated passion fruit drink.

**Figure 2:**
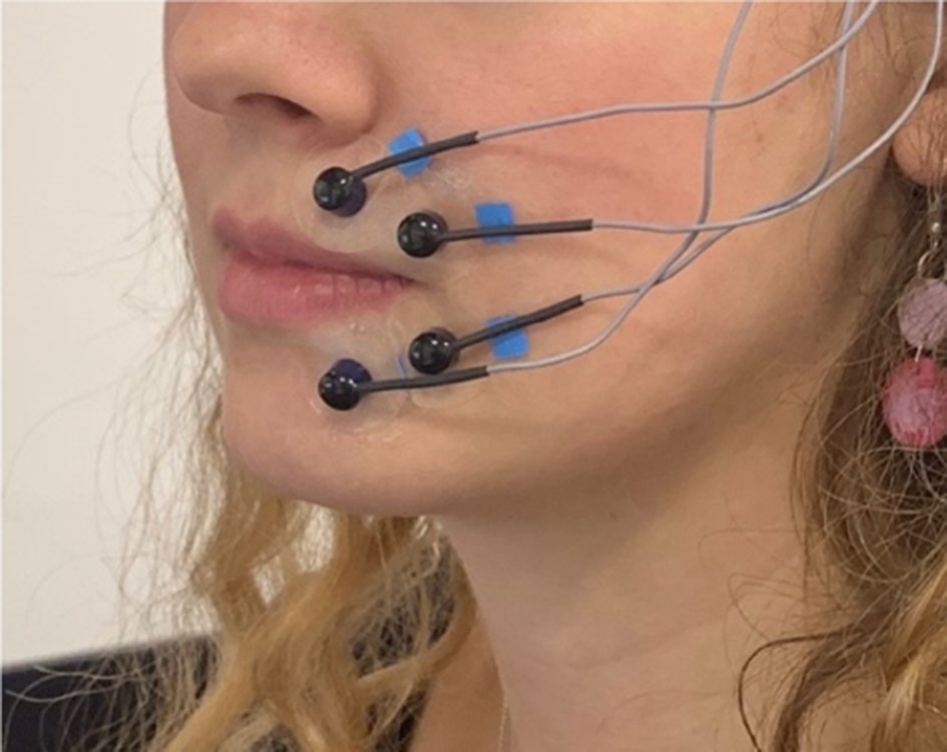
Placement of surface electrodes on the upper and lower lip, with the ground electrode on the temple, as used in Study 3.

Study 1 tested the effect of glass shape—straight-sided vs. outward-sloped tumblers (see Fig. 1)—on drinking rates. We used video recordings to extract relevant information, including the height of liquid remaining in the glass, which allowed us to model the pace of consumption over time—i.e., ‘drinking trajectory’—from the images. The experimental procedures have been used in previous studies also measuring the effect of glass shape on total drinking time^16,17^, and drinking trajectory^21^. Study 2 extended Study 1 by exploring the effect of glass shape on the volume consumed, using more extreme differences in glass shape—comprising straight-sided wine flutes vs. outward-sloped martini coupes (see Fig. 1). This experiment involved a bogus taste test in which participants were asked to drink as much or as little as they liked while rating the flavour of the drinks (as in ^27,28^). The bogus taste test has been validated and correlates with other measures of intake for alcohol^29^ and food^30^. Study 3 tested a novel potential mechanism for decreased consumption from straight-sided glasses, namely, the extent the lips are pursed during sipping—lip embouchure. We used facial electromyography (EMG) to detect the muscle activity of the upper and lower lips during sipping of soft drinks from straight-sided wine flutes and outward-sloped martini coupes (see Fig. 2 for placement of facial electrodes). Taken together, this set of studies provides evidence to advance our understanding both of the potential impact of glass shape on soft drink consumption, and ‘why’ it might be effective (i.e., through exploring mechanisms).

All studies (including planned analyses) were pre-registered on the Open Science Framework, where data and code is also available (https://osf.io/4tx3c/; https://osf.io/j9hqu/; https://osf.io/75b89/). Analyses were conducted in RStudio. For a summary of key outcome measures split by condition, see Table 1. Model diagnostics were checked and were all found to be satisfactory. Removing individuals who guessed the purpose of each study did not impact the findings (numbers of participants who guessed correctly, as follows: Study 1 (n = 9); Study 2 (n = 5); Study 3 (n = 3)). Video coding reliability was checked by an independent coder for Study 1 and 2 and was found to be satisfactory (see SI Appendix).

**Table 1:** Summary of key outcomes for Studies 1, 2 and 3.

		Glass shape	
		Straight-sided	Outward-sloped
Study 1			
Total drinking time (sec)^a^		299.96 (260.93, 344.83)	300.88 (259.87, 348.37)
Drinking trajectory (AUC)^bc^		0.506 (0.03)	0.630 (0.09)
Mean sip size (ml)^a^		22.61 (20.44, 25.01)	25.21 (22.76, 27.92)
Midpoint bias (ml)^b^		− 3.72 (15.53)	− 17.81 (17.06)
Study 2			
Amount consumed (ml)^b^		242.79 (113.60)	298.49 (156.69)
Midpoint bias (ml)^b^		− 4.60 (8.33)	− 6.00 (10.35)
Number of sips^b^		28.97 (12.53)	27.39 (11.15)
Mean sip size (ml)^b^		8.75 (3.47)	11.40 (5.45)
Study 3			
Lip pursing (%)—upper^d^		14.06 (13.33, 14.83)	12.91 (10.69, 15.59)
Lip pursing (%)—lower^d^		14.03 (13.40, 14.68)	11.68 (9.60, 14.20)
Sip size (ml)^d^		10.07 (9.60, 10.56)	12.16 (10.65, 13.89)

^a^Values are back transformed from log10 (Geometric mean and 95% CI), from unadjusted models.

^b^Values are unadjusted M (SD).

^c^AUC refers to the area under the curve, calculated from individual plots of amount consumed (%) over time (%). Larger AUCs indicate more decelerated drinking. Values of AUC taken from participants for whom all sips were recorded as volumes (n = 16).

^d^Values are back transformed from log (Geometric mean and 95% CI), from models adjusting only for repeated-measures.

## Results

## Study 1

### Overview

In Study 1, we sought to test whether drinking rates differed, depending on the glass being drunk from. We predicted that, in line with a previous study^,17^, soft drinks would be consumed more slowly from straight-sided glasses than outward-sloped ones. Additionally, we explored whether micro-drinking behaviours, including drinking trajectories—the pattern of consumption over time, mean sip size, mean sip duration, mean interval duration, as well as visual perception of drink midpoints, would differ.

We recruited 200 participants (50% female)—predominantly students and staff at the University of Cambridge, England—to take part in the study between May and July 2018. For more information on sample size determination and eligibility, see SI Appendix. Due to a video-recording malfunction, data from two participants (both female) were excluded from the analysis, leaving a total sample of N = 198. Baseline demographic and study-relevant characteristics are given in SI Appendix Table S1.

In a between-subjects design, participants were randomised (stratified by gender) to receive a soft drink in one of two glasses (straight-sided or outward-sloped tumbler). Participants consumed the drink at their own pace while watching a nature documentary (as in previous studies;^16,17^), and the primary outcome was total drinking time. Video recordings were taken during the experiment and subsequently coded for all drinking behaviours—total drinking time, drinking trajectory i.e., drinking pattern over time, mean sip size, mean interval duration, and mean sip duration. After finishing the drink, participants completed a midpoint estimation task by physically filling or emptying drinks from the same shape glass they had previously drunk from until the glass was perceived to be half-full (a task used in previous studies;^17,20^). Midpoint bias reflected estimations which deviated from the drink’s true halfway point (i.e., underfilling or overfilling the glass).

### Results

#### Total drinking time

Visual inspection of distributions indicated a positive skew for total drinking time. This was transformed using a log10 function to satisfy regression modelling assumptions. Back-transformed geometric means (geomeans) with 95% CIs are thus reported. Adjusting for pre-specified covariates—gender, thirst, and BMI—there was no evidence that total drinking time differed between glass shapes (0.26% faster from the straight glass than the outward glass; 95%CI: -21.4%, 18.1%, *p* = 0.979, see SI Appendix Table S2 for adjusted and unadjusted regression analyses).

#### Drinking trajectory

Drinking trajectories—drinking patterns over time—were compared between glass shapes. Drinking trajectory was determined by plotting estimates of volume remaining in the glass—which started at 330 ml and ended at 0 ml—over elapsed time. Time was standardized to represent the proportion of overall time taken, to account for differences between participants in total drinking times, and volume consumed was transformed to 0–100% cumulative intake.

For the great majority of participants (182/198), the volumes remaining in the glass were not recorded after every sip taken. This was due to not placing the glass on the table in-between sips, and/or holding the glass and occluding the liquid. These factors prevented the accurate measurement of heights of the liquid and glass to determine volume remaining after each sip. To deal with missing data, we excluded participants who had incomplete drinking trajectory data—leaving Subset A: participants for whom volume remaining was recorded after every sip (n = 16), and Subset B: participants for whom volume remaining was recorded after at least 50% of sips (n = 94). As a result, one limitation of the trajectory data is that the tendency of participants in these subsets to put down their glass may represent a particular style of drinking behaviour. For participant characteristics and outcome measures split by subset, see SI Appendix Table S3.

**Figure 3:**
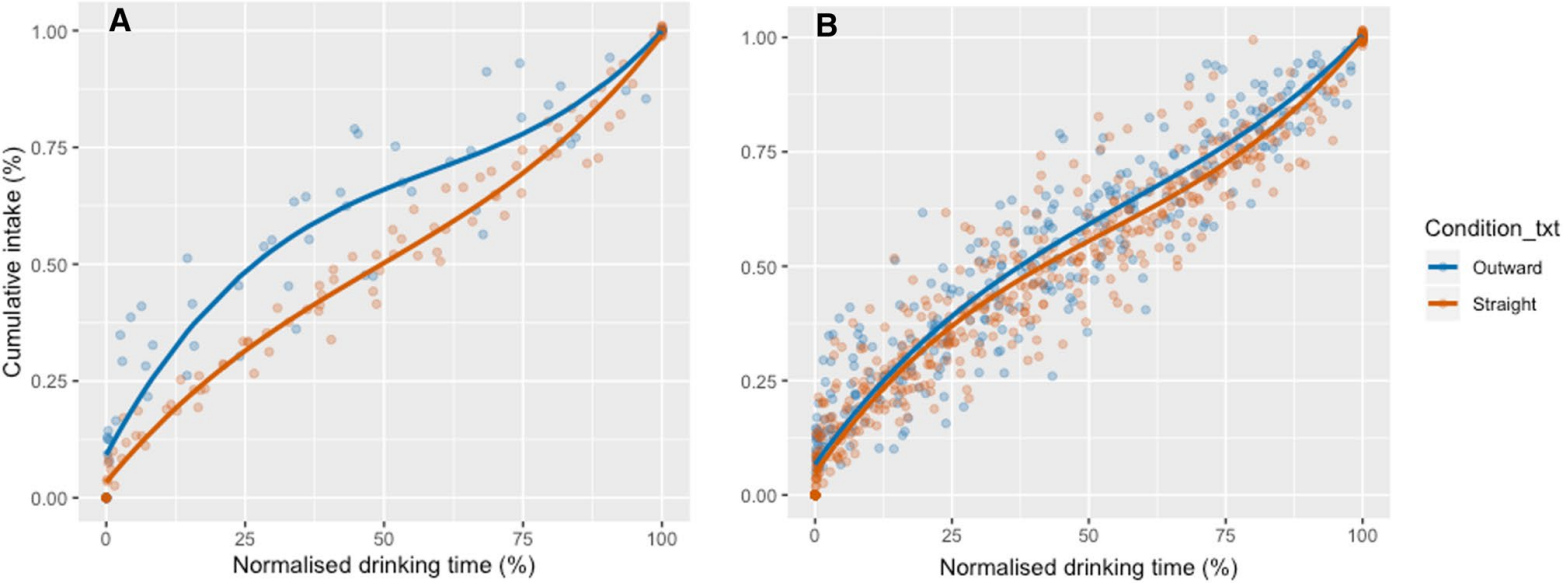
(**A**). Cumulative intake over time, Study 1. Lines indicate cubic curve fits. Participants with all sips recorded as volumes (n = 16). (**B)**. Cumulative intake over time, Study 1. Lines indicate cubic curve fits. Participants with at least 50% of sips recorded as volumes (n = 94).

For both subsets, a cubic (S-shaped) model had the lowest AIC, as compared to quadratic models (used to represent the drinking trajectory data in a previous paper—see^21^), for both outward and straight conditions, and thus was the best fit of the drinking trajectory data. Using Subset A, the cubic model predicted that at 50% time, 66.0% had been consumed from outward glasses (95% CI 59.6%, 72.7%), while 50.3% had been consumed from straight glasses (95% CI 47.5%, 52.7%), see Fig. 3A. Using Subset B, this model predicted that at 50% time, 59.2% had been consumed from the outward glasses (95% CI 56.8%, 61.8%), while 55.5% had been consumed from straight glasses (95% CI 53.4%, 57.8%), see Fig. 3B. Confidence intervals were calculated by bootstrapping.

The area under the drinking curves (AUC)—a proxy for drinking trajectory—was also calculated. Larger AUCs (i.e., closer to 1) are indicative of a more decelerated pattern (characterized by more consumed in the first half of the drinking period). AUCs were 24.6% larger for the outward-sloped glasses for Subset A (95%CI: 8.9%, 40.3%; *t*(8.264) = 3.60, *p* = 0.0067) and 6.3% larger for the outward-sloped glasses for Subset B (95%CI: 0.91%, 11.7%; *t*(85.06) = 2.32, *p* = 0.023), see SI Appendix Table S4 for average AUCs, by condition, for both subsets.

#### Sip size, sip duration, interval duration

Adjusting for the effects of gender on log mean sip size (B_Male_ = 0.15, *p* < 0.0001), sips were 11.1% larger from the outward-sloped glass than the straight glass, however the data were also consistent with smaller sips (95% CI − 2.8%, 27.0%), *p* = 0.123). Larger sips were associated with faster total drinking times, *r*(198) = -0.50, *p* < 0.0001). Gender did not significantly predict mean sip duration or mean interval duration, so was not included in these models. Sip durations did not differ between glass shapes (8.1% longer from outward-sloped glasses, 95% CI − 3.8%, 21.6%, *p* = 0.190). Interval durations did not differ between glass shapes (10.7% longer from outward-sloped glasses, 95% CI − 9.1%, 34.7%), *p* = 0.312).

#### Midpoint bias

Midpoints were underestimated from both glasses, consistent with under-filling the glass when estimating the halfway point. Individuals under-estimated the midpoints of outward-sloped glasses to a greater degree (mean difference = 14.1 ml, 95%CI: 9.5 ml, 18.7 ml), *t*(196) = 6.1, *p* < 0.0001). Midpoint bias was not associated with total drinking time, *r*(198) = -0.09, *p* = 0.196).

### Summary

There was no evidence that total drinking time differed depending on the glass being drunk from (failing to replicate a previous study^17^), though there was some evidence that drinking trajectories differed, with more decelerated drinking from outward-sloped glasses—characterised by a greater amount consumed in the first half of the drinking episode—and more linear drinking from straight-sided glasses. While this indicates that straight-sided glasses may lead to a different pace of drinking, it remains to be seen whether glass shape influences the amount that is consumed. As in previous research, there was evidence of midpoint bias, with midpoints underestimated more from outward-sloped glasses^16,17,20^. However, there was no evidence that this bias in perception of drink midpoints was associated with drinking time.

## Study 2

### Overview

In Study 2, we sought to extend Study 1 by investigating whether glass shape would influence amount of a soft drink consumed. In particular, we predicted that straight-sided glasses would lead to less overall consumed than outward-sloped ones. As in Study 1, we also compared micro-drinking behaviours (number of sips) and visual perception of drink volumes (midpoint bias).

We recruited 72 (50% female) participants—predominantly students and staff at the University of Cambridge, England—to take part in this study between February and March 2019. This study used an adaptive design with an internal pilot^31-33^. For more information on the stopping rules for recruitment, see SI Appendix. Baseline characteristics are shown in SI Appendix Table S5. Due to video-recording malfunction, data for number of sips was only available for 71/72 participants.

In a between-subjects design, participants were randomised to one of two conditions (stratified by gender), receiving four soft drinks to taste and rate (each 165 ml), served in either straight-sided wine flutes or outward-sloped martini coupes (see Fig. 1. for images of the glasses). The primary outcome was amount consumed during a 10-min bogus taste test (a validated measure of intake;^29^). Secondary outcome measures were number of sips (determined from video recordings which were subsequently coded for sips), and midpoint bias (assessed after the taste test, in the same way as in Study 1).

### Results

#### Amount consumed

Visual inspection indicated no evidence of skew in the primary outcome measure: volume consumed (ml). Adjusting for pre-specified covariates (gender, thirst, drink enjoyment and maximum oral capacity), our model indicated that 72.1 ml less was consumed from straight-sided glasses than outward-sloped glasses (95% CI − 11.7 ml, − 132.6 ml), *p* = 0.022 (see SI Appendix Table S6 for full unadjusted and adjusted linear models).

#### Number of sips

There was no evidence that total number of sips differed between glass shapes (mean difference = − 1.6 sips from the outward-sloped glass, 95% CI − 7.2, 4.0), *p* = 0.58. Number of sips was positively associated with total amount consumed, with fewer sips associated with less being consumed overall, *r*(71) = 0.48, *p* < 0.0001. Mean sip size—though not pre-specified—was also explored, to account for differences in amount consumed. This was calculated by dividing total volume consumed by number of sips. Mean sip sizes were 2.7 ml smaller from straight-sided glasses than from outward-sloped glasses (95% CI 0.48 ml, 4.8 ml), *p* = 0.017. Mean sip size was also strongly and positively associated with total amount consumed, with smaller mean sips associated with less consumed overall, *r*(71) = 0.67, *p* < 0.0001.

#### Midpoint bias

Drink midpoints were underestimated for both glasses. Though outward-sloped glasses led to lower midpoint estimates than straight-sided glasses, there was no evidence that the difference in midpoint bias was meaningful (mean difference = − 1.4 ml, 95% CI − 5.8 ml, 3.0 ml), *t*(68) = − 0.63, *p* = 0.531). Midpoint bias was not associated with amount consumed, *r*(72) = − 0.03, *p* = 0.827.

### Summary

Consistent with our predictions, Study 2 demonstrated that, in a bogus taste test paradigm, less was consumed from straight-sided glasses than outward-sloped ones, this time using stemmed glasses rather than tumblers, and using a non-carbonated soft drink. Lower number of sips and smaller average sips size were associated with less consumed overall. Midpoint bias was not associated with amount consumed.

## Study 3

### Overview

In Study 3, we sought to investigate a potential mechanism for reduced intake from straight-sided glasses—namely, the extent lips are pursed during sipping, or lip embouchures. We predicted that when sipping from straight-sided glasses, lips would be more pursed (characterised by higher levels of muscle activity in the upper and lower lips, measured using surface EMG) than when sipping from outward-sloped ones. We also measured sip sizes in real time, to explore whether glass shape influenced sip size, and whether embouchure mediated effects of glass shape on sip size.

We recruited 40 females—predominantly students and staff at Macquarie University—to take part in an experiment on campus at Macquarie University, Australia, between June and July 2019. For more information on sample size determination, see SI Appendix. For demographic and study-relevant information see SI Appendix Table S7.

In a within-subjects design, participants were served four soft drinks (165 ml each), in two straight-sided glasses (wine flutes) (A), and two outward-sloped glasses (martini coupes) (B), in the sequence ABAB or BABA, with order randomised. Three sips were taken from each drink. Sip volumes were recorded after each sip, using a concealed weighing scale. The study used an adapted bogus taste test with the addition of surface facial electrodes placed on the upper and lower lips, which measured muscle activity, as a proxy for embouchure. The primary outcome measure was lip muscle activity (co-primary endpoints of upper and lower lip muscle activity) which was transformed and expressed as a percentage of each individual’s maximal voluntary contraction (%MVC). Maximal voluntary contraction was measured by asking participants to protrude their lips as firmly as possible. The secondary outcome measure was sip size (ml).

Visual inspection of distributions indicated positive skew for embouchure (%MVC upper, %MVC lower) and sip size (ml). All three variables were transformed using a log function, improving the shapes of the distributions. Back-transformed geomeans with 95% CIs are reported in Table 1 for these outcome measures, adjusting only for repeated-measures.

### Results

#### Embouchure

Two linear mixed effects models were used, predicting the co-primary endpoints of i) upper and ii) lower lip muscle activity (log %MVC) from glass shape, adjusting for standard crossover design variables (treatment: glass shape, sequence: ABAB/BABA, and time period: drink number) as well as sip number (1st, 2nd, 3rd) and with participant as a random effect. These models indicated that, when sipping from straight-sided glasses, participants used 8.9% more upper lip muscle activity (95% CI 3.3–14.8%), *p* = 0.0017, and 20.03% more lower lip muscle (95% CI 14.7%, 25.6%), *p* < 0.0001, than when sipping from outward-sloped glasses. Findings were significant after adjusting for co-primary endpoints (i.e., significance when alpha < 2.5% using Bonferroni adjustment). See SI Appendix Table S8 for full models.

#### Sip size

Two linear mixed effects models were used, predicting log sip size from upper and lower muscle activity (%MVC), adjusting for the same variables as above, as well as baseline thirst, and maximum oral capacity, and with participant as a random effect. These models indicated that glass shape predicted sip size, with sips that were 17.3% smaller and 16.6% smaller from straight-sided glasses than outward-sloped glasses, when adjusting for upper %MVC (95% CI 13.3%, 21.3%, *p* < 0.0001) and lower %MVC (95% CI 12.2%, 20.8%, *p* < 0.0001), respectively. However, there was no evidence that muscle activity predicted sip size in these models: for every 1% increase in upper lip muscle activity used, there was a 0.19% decrease in sip size (95% CI − 0.56, 0.2), *p* = 0.337, and for every 1% increase in lower lip muscle activity used, there was a 0.36% decrease in sip size (95% CI − 0.95, 0.3), *p* = 0.251. See Table S9 for full models.

In exploratory analyses, we removed glass shape from these models to determine whether lip muscle activity predicted sip size for upper and lower %MVC respectively, without adjusting for the effects of glass shape on sip size. In this analysis, lower lip muscle activity (%MVC lower) did predict sip size, such that for every 1% increase in lower lip muscle activity used, there was a 1.13% decrease in sip size (95% CI − 1.70, − 0.51), *p* = 0.0002. There was no evidence that upper lip muscle activity (%MVC upper) predicted sip size; for every 1% increase in upper lip activity used, there was a 0.27% decrease in sip size (95% CI − 0.66, 0.14), *p* = 0.191.

### Summary

In line with predictions, we found that lips were more pursed when participants sipped from straight-sided glasses, as compared to outward-sloped glasses. Correspondingly, sips were smaller from straight-sided glasses, and there was some evidence of an association between lip muscle activity and sip size (with smaller sips associated with more pursed lower lips).

## Discussion

The studies reported here investigated the effects of varying glass shape—straight-sided vs*.* outward-sloped—on the consumption of soft drinks, using diverse techniques to measure drinking behaviours. In Study 1, though the overall time spent drinking was equivalent for those drinking from straight-sided and outward-sloped glasses (failing to replicate a previous study^17^), when exploring the micro-drinking behaviours in detail, we found that drinking trajectories differed. In particular, drinking from outward-sloped glasses was characterised by a more decelerated pace, a similar finding to previous research^21^, which found more decelerated drinking from short-wide glasses than tall-narrow ones. In Study 2, using a bogus taste test paradigm, those tasting and rating drinks served in straight-sided glasses consumed less overall than those drinking from outward-sloped ones. Average sip sizes were also smaller from straight-sided glasses, and there was an association between sip size and total amount consumed. In Study 3, using an adapted bogus taste test paradigm with sips taken in regimented stages to allow for the measurement of lip muscle activity, lips appear to be more pursed when drinking from straight-sided flutes. The same study showed a corresponding effect of glass shape on sip size—with smaller sips taken from straight-sided flutes—and preliminary evidence that embouchure may be associated with sip size—for lower lip embouchure, at least. To our knowledge, this final study is the first to use facial EMG to explore lip embouchures during sipping, a potential mechanism for reduced consumption from straight-sided glasses.

The three studies thus report broadly consistent findings that together suggest that glass shape influences drinking behaviours, including volume consumed. As discussed, one potential mechanism that may underlie some of the differences in drinking behaviour is the extent of lip pursing—or embouchure—during sipping. Affordance of micro-drinking behaviours—such as sip size via embouchures—seems a good candidate mechanism for explaining, at least in part, the reduction of intake from straight-sided glasses. An additional mechanism that we explored involved biases in visual perception of drink volumes. As in previous studies^16,17,20^, drink midpoints were underestimated more from outward-sloped than straight-sided tumblers in Study 1, indicative of midpoint bias from outward-sloped glasses. However, there was no evidence of a difference in midpoint bias in Study 2 using flutes and coupes (though the direction of the effect was consistent, and this adaptive design may have lacked power to detect small effects). Importantly, however, there was no evidence that midpoint bias was associated with drinking behaviour, including drinking time (in Study 1) or amount consumed (in Study 2). This suggests that—while sometimes present—biases in visual perceptions of drink volumes may not consistently influence drinking behaviour, and that other factors may better explain the effects of glass shape on drinking time and consumption.

The strength of this research lies in its novelty and scientific rigour: it is the first comprehensive set of studies focused on the impact of glass shape on drinking behaviour, measured using different study paradigms. The key findings are largely consistent across study paradigms testing different primary outcomes, including a novel physiological measure, and across different study designs—i.e.*,* both within- and between-subjects. Regardless of the exact glassware used—i.e.*,* tumblers in Study 1 and stemmed glasses in Study 2 and 3—or type of drink served—i.e., carbonated in Study1, non-carbonated in Studies 2 and 3—the findings suggest that serving drinks in straight-sided glasses is a potentially effective way to reduce consumption of drinks which harm health.

The studies also had a number of limitations worth noting. First, it remains unclear which exact features gave rise to the effects—outward-sloped glasses having wider rim apertures than narrower straight-sided ones. The latter two studies used more ‘extreme’ versions of the Study 1 tumblers, with rim diameters which varied more drastically. Rim diameter may have contributed to the observed effects on drinking behaviours, in addition to, or independently from, the effect of glass wall slope. Second, the study procedures may have failed to reflect real life drinking scenarios, limiting the ecological validity of the findings. Laboratory settings often lack certain characteristics that mark ‘real-life’ drinking environments—for example, the absence of drinking companions and the artificial nature of the tasks. There is also a need to examine drinking behaviours when people consume multiple drinks, to ascertain whether behaviour change persists after consuming a single drink. Study 2 went some way to address this, using a bogus taste test paradigm which involved tasting multiple drinks. However, while bogus taste tests are sensitive to desire to consume, comprising a valid measure of ad libitum consumption^29^, they do not offer insight into how drinking might be impacted over a longer drinking session when multiple drinks might be fully consumed (i.e., not tasted). Finally, though Study 1 utilised a more granular approach to measure drinking behaviours by measuring cumulative intake over time, based on detecting volume consumed from images (a similar method to^21^), one limitation relates to attrition of data for this analysis. Given the nature of the task, there was often occlusion of liquid in the images of participants drinking, sometimes due to participants holding the glass at an angle, or covering the liquid with their hands. This occlusion in the images prevented the accurate measurement of the liquid:glass heights and thus the calculation of volumes. As such, there were only sixteen participants for whom all sips were recorded as volumes (Subset A). Excluding participants for whom < 50% of sips were recorded as volumes (Subset B) showed similar patterns of findings, though the effects were smaller.

Future studies might build on this set of findings in a number of ways. First, as discussed, studies are required in real-world drinking environments, which will also enable effect size estimates. This might include studies set in pubs, bars and restaurants, comparing the volume purchased and consumed according to the glasses drinks are served in, as has been investigated for wine glass size;^13-15,34^. Relatedly, while developing novel interventions to reduce consumption of health-harming drinks remains important, it is worth investigating whether the converse is true—that is, whether outward-sloped glasses may help to increase consumption of healthy drinks, relative to straight-sided glasses. Thus, future studies could explore the impact of glass shape on water consumption in medical settings where hydration is warranted. Second, research is required to ascertain the parameters of these effects and in particular, whether the effects apply to alcoholic drinks as well as to soft drinks. This is worth exploring given that a previous study found evidence for slower drinking from straight-sided glasses than outward-sloped glasses, but only for beer, and not for a soft drink^16^. It is therefore possible that the effects found in the present studies could be stronger for alcohol. Third, though this set of studies has explored two potential mechanisms for the effects of glass shape on consumption—via biases in visual perceptions of glass midpoints and via affordance of embouchures and their potential impact on micro-drinking behaviours—future studies might usefully identify additional mechanisms. Understanding the processes through which glass shape influences consumption will advance our overall understanding of the effects and in turn lead to optimising glassware design for more effective, more cost-effective, and more targeted interventions.

This set of studies contributes to a small but growing evidence base of a potentially effective, easily implemented, small-scale physical environment intervention^9,10^ for reducing consumption of sugar-sweetened beverages. In combination with other behaviour change strategies, adopting straight-sided glasses may prove to be one intervention contributing to the many needed to reduce consumption of drinks that harm health.

## Methods

Written informed consent was obtained for all participants at the start of each study, and approvals were obtained from the University of Cambridge Psychology Research Ethics Committee (Study 1: PRE.2018.015, Study 2: PRE.2018.122, Study 3: PRE.2019.030), and the Macquarie University Research Ethics Committee (Study 3: 5201954159069). All studies were conducted in accordance with the relevant institutional guidelines and regulations. More information (on eligibility and recruitment for each study), is given in SI Appendix. Pre-registered study protocols are available on the OSF (https://osf.io/4tx3c/; https://osf.io/j9hqu/; https://osf.io/75b89/). Written informed consent was obtained from the participant for publication of their image (Fig. 2.) in an online, open access scientific paper.

## Study 1

### Measures

Drinking behaviours were measured by coding video recordings. The start and end of each sip was coded using a key press, giving total drinking time (the primary outcome measure), mean sip duration, mean interval duration, and total number of sips. To determine mean sip size, we divided the total amount consumed—330 ml—by the total number of sips.

To determine drinking trajectory, we first created models predicting volume from the height of known increments of liquid relative to the height of the glass, for each of the glass shapes. These models allowed us to estimate the volume remaining in the glass when it was placed on the table in the study videos (as long as there was only minimal occlusion to the liquid). We then used these estimates to plot each individual’s cumulative intake (%) over time (%). To determine drinking trajectory, we combined the plots and fitted models by condition. Using these models, we calculated amount consumed at 50% time. We also calculated the areas under each individual’s drinking curve, as a proxy for trajectory (higher scores are indicative of more decelerated drinking). Due to the need for glasses to be placed on the table with minimal occlusion to the liquid, trajectories were only able to be calculated for subsets of participants—those who regularly placed their glass down (see SI Appendix Table S3 for characteristics and outcomes of these subsets). Given that analyses were conducted on two subsets of data (Subset A = 100% of sips recorded as volumes, Subset B =  > 50% of sips recorded as volumes), we applied the conservative Bonferroni adjustment to the threshold of significance (i.e. *p* < 2.5%).

Midpoint bias was assessed using a task involving trials of filling or emptying drinks from the allocated glass until the glass was perceived to be half-full (as in previous studies^17,20^). The six poured estimates of the midpoint of the drink were averaged to provide a single estimate for each participant. This was then subtracted from 165 ml (the true midpoint), to determine midpoint bias. Negative values reflect underestimation of the true midpoint (pouring too little liquid into the glass), while positive values indicate overestimation of the true midpoint (pouring too much liquid into the glass).

### Procedure

Participants attended a single session ostensibly exploring ‘the impact of glucose on cognitive performance’. On arrival, participants completed eligibility screening, and stated their age, gender, height, weight, and thirst (1–10).

A 330 ml can of chilled Appletiser was served in the appropriate glass (based on randomisation). Participants were asked to consume the drink at their own pace, whilst watching a documentary. Video recordings were taken during this time.

Participants then completed two filler tasks (word search and drink ratings). Finally, they estimated the midpoint of their drink. A full portion was placed in front of participants (in the glass they drank from previously), and they were asked to pour estimates of the halfway point of the drink (i.e., 165 ml). There were six estimates in total (three from glass to jug, and three from jug to glass). Participants were invited to use a ‘Reference Glass’ throughout the task to aid accuracy, which contained the full 330 ml portion.

Finally, participants were asked what they thought the purpose of the study was, to examine the effectiveness of the cover story in blinding participants to the true nature of the study. Participants were paid £7 for their time and expenses, and told they would receive a full study debrief via email once all participants had taken part.

## Study 2

### Measures

Total amount consumed (ml) —the primary outcome measure—was measured by weighing the four glasses before and after the 10-min taste test, using high precision scales. The weights of the glasses after consumption were subtracted from the initial weights to determine the total volume consumed.

Micro-drinking behaviours (i.e., number of sips) were measured by coding the video recordings taken during the drinking sessions (with each sip initiation and endpoint coded by a key press, as in Study 1). Midpoint bias was measured in the same way as Study 1 (with the true midpoint of these drinks being 82.5 ml).

Maximum oral capacity was measured—to adjust for in the primary analysis—and calculated by asking participants to fill their mouth to full capacity from a cup filled with room temperature water, before spitting into an empty jug (weighed before and after to determine total volume). This task completed twice (with the total volume then divided by two), and was disguised as a ‘palate cleanser’. Drink enjoyment was also measured—to adjust for in the primary analysis—from ratings given during the taste test, with scores for ‘tasty’ and ‘pleasant’ (rated from 1–10) averaged for all four drinks.

### Procedure

Eligible participants attended a single session to take part in a study on ‘taste preferences’. On arrival, participants completed eligibility screening, and demographic questions, including age, gender, height, weight, and thirst (1–10). Next, participants completed the ‘palate cleanser’—twice filling their mouth with water and spitting into a jug to determine oral capacity.

For the taste test, the experimenter prepared four drinks—a total of 660 ml (divided into four 165 ml portions) of Teisseire Passion Fruit Le Sirop (1 part syrup, 7 parts water), served in either straight-sided stemmed wine flutes, or outward-sloped stemmed martini coupes, depending on randomisation. The drinks were placed on mats labelled A, B, C, and D. Participants were told to taste and rate the drinks according to 10 descriptors (fruity, smooth, sweet, refreshing, bitter, strong-tasting, gassy, pleasant, light, and tasty; see^27^), for 10 min. As well as completing these ratings, participants were be asked to indicate their preferences from 1(favourite)—4(least favourite), to reinforce the cover story. They were told to drink as much or as little of the drinks as they would like, to assist their ratings.

After the 10-min taste test, the experimenter returned and he participant completed the midpoint pouring task (involving six poured estimates of 82.5 ml). The procedure was the same as for Study 1. Finally, the participant was asked to guess the purpose of the study, using an open-ended question presented on screen. As in Study 1, participants were paid £7 for their time and expenses, and received a full study debrief via email once all participants had taken part. After the participant had left, the experimenter weighed the glasses to determine volume consumed.

## Study 3

### Measures

The primary outcome measure in this study was embouchure (%MVC). Embouchure was measured using surface electrodes which detected the amplitude of activity in the upper and lower lips. Higher amplitudes of lip muscle activity are a proxy for a more ‘pursed’ embouchure. A two-channel Biopac MP160 system was used for continuous electromyographic (EMG) signal acquisition, and data inputs were recorded and processed using *AcqKnowledge* 5.0 software (BIOPAC systems Inc., USA). Raw amplitudes of activity were transformed using a script which generates a rectified and integrated copy of the data over a period of 250 ms and then rescales the channel so that this runs from 0–100%, with an individual’s maximum voluntary contraction representing 100% (as recommended for facial EMG measurement^35^. To determine each individual’s maximum voluntary contraction, maximal lip compression trials were run, involving participants protruding their lips as hard as possible three times (as in a previous study^26^). The signal with the highest amplitude (from the three maximum lip compression attempts) was selected to be used for standardizing by each participant’s maximum voluntary contraction.

The secondary outcome measure was sip size (ml). Individual sip sizes (ml) were measured in real time, for each of the three sips taken from each of the four glasses. Sip sizes were noted by the experimenter after each sip, when the glass was placed on concealed weighing scales. The glass was weighed before consumption (i.e., with 165 ml drink inside) and subsequently after each sip was taken during the taste test.

Maximum oral capacity was also measured—to adjust for in the primary analysis—in the same way as Study 2 (i.e., disguised as a palate cleanser).

### Procedure

Participants were invited to attend a single study session investigating ‘reactions to drinks served in different containers’. On arrival, participants completed eligibility screening, followed by the ‘palate cleanser’ task. The participants answered baseline demographic questions, and rated their thirst (1–10).

To prepare the skin for electrode placement, it was wiped with make-up remover, and brushed with an exfoliative pad and a small amount of abrasive gel (NuPrep). The four surface electrodes were then filled with electrode gel (SignaGel), and affixed to the upper and lower lips on the left side (see Fig. 2.) using adhesive discs. For the lower left quadrant, one electrode was placed 1 cm below the cheilion (corner of mouth), and the paired electrode placed 1 cm medial and slightly below (corresponding to the edge of the mouth). The upper left quadrant followed the same pattern (see^22,35^ for recommended placement of electrodes for facial EMG). The ground electrode was placed on the temple. Trailing wires were clipped behind the ear. An Impedance checker was used to assess signal conductivity. If impedance was greater than 30 kO the corresponding electrodes would be re-affixed.

Participants were served a total of 660 ml (divided into four 165 ml portions) of Teisseire Passion Fruit Le Sirop (diluted 1 part syrup, 7 parts water), in two straight-sided stemmed wine flutes, and two outward-sloped stemmed martini coupes, in the order assigned to them.

Participants were instructed to take three sips taken from each drink. Each sip was divided into four stages (adapted from a previous study^26^). The four stages were indicated on the screen, as follows.

Step 1— “Relax”: “Please relax, keeping your lips at rest”.

Step 2— “Prepare”: “Please lift the glass and hold it in front of your mouth”.

Step 3— “Remove”: “Now raise the glass to your lips and take a sip”.

Step 4— “Swallow”: “Swallow the sip in one swallow”.

Participants first practised these stages with a glass of water. They would next begin the taste test, taking three sips from each glass. After taking three sips from the drink, the participant rated the drink on the same 10 descriptors as Study 2. They were also asked to rate, from 1–10, how much they enjoyed the experience of consuming their drink from that glass. All of these questions were filler questions, to add to the cover story and disguise the true aim of the study. Once all four drinks had been tasted and rated, the participant was asked to complete the maximal lip compression tasks. They were asked to “squeeze your lips together as hard as you can, so they protrude as far as possible”. They did this 3 times, as prompted by on-screen instructions. After electrodes were removed, participants were asked to describe what they believed to be the purpose of the study. Participants were paid $10 for their time and expenses, and told they would receive a full study debrief via email once all participants had taken part.

## Supplementary information

Supplementary file1.
