# Supplementary material for: Glass shape influences drinking behaviours in three laboratory experiments
Source: Sci Rep. 2020 Aug 7;10:13362. doi: 10.1038/s41598-020-70278-6 (PMC7414130; doi:10.1038/s41598-020-70278-6)
Supplement: Supplementary file 1 — Supplementary file1. [file 41598_2020_70278_MOESM1_ESM.pdf]

Supplementary Information for

Glass shape influences drinking behaviours in three laboratory experiments

Tess Langfield*, Rachel Pechey, Philippe T. Gilchrist, Mark Pilling, Theresa M. Marteau.

* To whom correspondence may be addressed. Email: tess.ir.langfield@gmail.com.

This file includes:

Supplementary Text
Tables S1 to S9

Supplementary Text

Participants and sample size determination

Study 1

Participants were recruited from the students and staff at the University of Cambridge (UK), using mailing lists, flyers, and word of mouth. Eligibility was assessed at the start of the study session, and required that individuals: had not taken part in our previous study [17]; were over 18 years old; were in good physical health; had English as a first language or an equivalent level of fluency; were prepared to consume a drink that contains sugar; had no known allergies to any ingredients in Appletiser.

A power calculation with an alpha level of 0.05, power of 80%, and an effect size of 0.372 (observed in the previous study, when comparing total drinking time between straight-sided vs outward-sloped glasses; [17]) indicated that we would need a sample of $N = 182$ (91 per group). To allow for possible attrition due to video equipment malfunction, a sample size of $N = 200$ (100 per group) was used. The study sample size was pre-specified in our pre-registered study protocol (https://osf.io/4tx3c/).

Study 2

Participants were recruited from students and staff at the University of Cambridge (UK), using the same recruitment methods as Study 1. Eligibility criteria was the same as Study 1, though this study required participants to have no known allergies to any of the following ingredients: Fruit Juices from Concentrate 38% (20% Passion Fruit, 18% Lemon), Glucose-Fructose Syrup, Flavourings, Colours: Lutein, Paprika Extract, Stabiliser: E445.

The study sample size (N = 72, 50% female) was primarily driven by pragmatic considerations *i.e.* time and cost, is in line with convention (28) for an 'internal pilot', forming part of an adaptive design [31-32], and was pre-specified in the pre-registered study protocol (https://osf.io/j9hqu/). Given that previous studies had largely focused on the effects of glass shape on speed rather than consumption, we did not know what the effect size on consumption could be. As such, this internal pilot provided the necessary parameters (effect size and p value) for a sample size calculation. This adaptive design used the following stopping rules for recruitment at the interim analysis stage after outcome data had been collected on 72 participants.

1. If the effect of glass shape is significant at $p < 0.3\%$ for the primary interim analysis, or if no additional recruitment was required to achieve p < 4.7% for the primary final analysis (after sample size re-estimation), further recruitment would not be required. This is the *efficacy (utility) stopping criterion* (O'Brien-Fleming boundary).
2. If the sample size required to demonstrate $p < 4.7\%$ at the primary final analysis is unfeasible (with feasibility based on time and cost, being set at n >150 additional participants required) the trial will be stopped. This is the *futility stopping criterion*.

Together, these criteria represented early stopping rules. We stopped the trial after the internal pilot (N = 72) based on the utility stopping criterion, as our internal pilot analysis was already sufficient to detect an effect for the primary final analysis, at $p < 4.7\%$.

Study 3

Participants were recruited from the students and staff at Macquarie University (Australia), using the same recruitment methods as Studies 1 and 2. Eligibility criteria required that individuals: were female (given problems associated with detecting lip muscle activity in males with extensive facial hair); were over 18 years old; had not eaten, drunk (including water), or smoked anything for 30 minutes prior to the test session; had had no surgical procedures performed on the lips (e.g. cleft lip surgery or cosmetic reconstruction); did not have highly sensitive skin; were prepared to remove facial make up; were prepared to consume drinks that contain sugar; and had no known allergies to any of the following ingredients: Fruit Juices from Concentrate 38% (20% Passion Fruit, 18% Lemon), Glucose-Fructose Syrup, Flavourings, Colours: Lutein, Paprika Extract, Stabiliser: E445.

The sample size (N = 40) was based on pragmatic constraints – i.e. time and cost, was pre-specified in our pre-registered study protocol (https://osf.io/75b89/), and is in line with previous studies [36-38].

Sensitivity analyses

Study 1
Individuals (n = 9) who correctly guessed the purpose of the study and/or the aims of the drinking task (to measure drinking speed) were excluded from the analysis. Removing these data had no impact on results from the primary or secondary analyses.

Study 2
Removing individuals (n = 5) who correctly guessed the purpose of the taste test (i.e. to measure volume consumed) had no impact on the primary or secondary analyses.

Study 3
Removing individuals (n = 3) who correctly guessed the true aims of the study did not impact the primary or secondary analyses.

Reliability analyses

Reliability analyses were conducted for Study 1 and 2, given the involvement of coding videos of participant behaviour.

Study 1
Video coding was assessed for reliability, for all video data (total drinking time, mean sip size, mean sip duration, mean interval duration, and volume over time data). For volume over time data (drinking trajectories and AUC), an independent coder measured glass:drink height ratios from the videos of 10 participants (40 observations). The 40 height ratios produced by the coder were then compared with the equivalent height ratios extracted for the primary data.

Video coding was consistent across video coders, with high inter-rater reliability, assessed using single measures intra-class correlation. Strong positive associations were observed for total drinking time (20) > 0.99, $p < .0001$, mean sip size (20) = 1.00, $p < .0001$, mean sip duration (20) = .97, $p <.0001$, mean interval duration (20) > 0.99, $p < .0001$, and height ratios (40) > 0.99, $p < .0001$.

Study 2
Inter-rater video coding reliability was assessed for 10 videos. The recorded number of sips coded from each of the 10 participants was identical.

Table S1.

Study 1 - Baseline characteristics of participants by condition.

	**Straight-sided (n = 100)**	**Outward-sloped (n = 98)**	**Overall (N = 198)**
**Gender (%)**			
Female	50.0	49.0	49.5
Male	50.0	51.0	50.5
**Highest Educational Qualification (%)**			
GCSE/O Level	1.0	2.0	1.5
AS/A Level	37.0	32.7	34.8
Undergraduate degree	35.0	47.9	41.4
Postgraduate degree	27.0	17.3	22.2
**Age (years)**	22 (20,26)	22 (21, 24.75)	22 (20,26)
**Thirst (1-10)**	6.09 (1.64)	6.16 (1.54)	6.13 (1.59)
**BMI (kg/m2)**	22.76 (3.14)	22.75 (3.49)	22.75 (3.31)

Notes. Thirst was rated from 1 (“Not at all”) to 10 (“Extremely”). Age is median (IQR); BMI and thirst are mean (SD). Four individuals in the outward-sloped condition opted to omit information on BMI.

Table S2.

Study 1 - Unadjusted (univariate) and adjusted (multivariate) regression, predicting log10(total drinking time).

	**Unadjusted regression analyses**					**Adjusted regression analyses**			
Independent variable	*B*	Exp(*B*)	95% CI Exp(*B*)	*p*-value	R^2	*B*	Exp(*B*)	95% CI Exp(*B*)	*p*-value
(Intercept)	-	-	-	-	-	2.762	577.70	271.96 to 1,227.12	< .0001
**Glass shape**					.00				
Straight-sided	-0.001	0.997	0.815 to 1.219	.976		-0.001	0.997	0.819 to 1.214	0.979
**Gender**					.075				
Male	-0.174	0.669	0.552 to 0.812	.0001		-0.174	0.669	0.548 to 0.817	.0001
**Thirst (1-10)**	-0.0013	0.997	0.936 to 1.062	.923	.00	-0.0076	0.983	0.922 to 1.047	.587
**BMI (kg/m^2)**	-0.010	0.977	0.947 to 1.007	.130	.0067	-0.0066	0.985	0.955 to 1.015	.325

Notes. Reference for Glass shape is Outward-sloped. Reference for Gender is Female. Thirst was rated from 1 (“Not at all”) to 10 (“Extremely”). Adjusted analyses: $F(4,189) = 4.547$, $p = .0016$, $R^2 = .0685$. Exp = Power of 10.

Table S3.

Study 1 - Baseline characteristics of participants and drinking behaviours, for full sample, and Subset A (100% of sips recorded as volumes) and Subset B (>50% of sips recorded as volumes), used for drinking trajectory analyses.

	**Full dataset (N = 198)**	**Subset A (n = 16)**	**Subset B (n = 94)**
**Condition (%)**			
Straight	50.5	50	54.2
Outward	49.5	50	45.7
**Gender (%)**			
Female	49.5	37.5	43.6
Male	50.5	62.5	56.4
**Highest Educational Qualification (%)**			
GCSE/O Level	1.5	0.0	1.1
AS/A Level	34.8	37.5	33.0
Undergraduate	41.4	50.0	43.6
Postgraduate	22.2	12.5	22.3
**Age (years)**	22 (20, 26)	22 (20.75, 26.25)	22.50 (21, 26.75)
**Thirst (1-10)**	6.13 (1.59)	6.06 (1.39)	6.14 (1.60)
**BMI (kg/m^2)**	22.75 (3.31)	21.89 (2.27)	22.83 (3.23)
**Total drinking time (min)**[a]	300.61 (271.64, 331.89)	351.56 (235.50, 523.60)	380.19 (331.89, 435.51)
**Mean sip size (ml)**[a]	23.88 (22.23, 25.64)	38.19 (30.69, 47.42)	27.93 (25.53, 30.55)
**Mean sip duration (sec)**[a]	1.90 (1.79, 2.02)	2.33 (1.77, 3.07)	1.99 (1.83, 2.17)
**Mean interval duration (sec)**[a]	19.28 (17.46, 21.28)	37.33 (24.72, 56.36)	29.72 (26.24, 33.65)

Notes. Thirst was rated from 1 (“Not at all”) to 10 (“Extremely”). Age is median (IQR); BMI and thirst are mean (SD).

a. values are back transformed from log10 (Geometric mean and 95% CI).

Table S4.

Study 1 - Impact of glass shape on drinking trajectory and other outcome measures, for Subset A (100% of sips recorded as volumes) and Subset B (>50% of sips recorded as volumes)

		Subset A (n = 16)		Subset B (n = 94)	
	**Glass shape**	**Straight**	**Outward**	**Straight**	**Outward**
**Drinking trajectory**	AUC	0.51 (0.03)	0.63 (0.09)	0.55 (0.07)	0.58 (0.07)
	Percentage consumed at 50% time	50.3 (47.5, 52.7)	66.0 (59.6, 72.7)	55.0 (53.4, 57.8)	59.2 (56.8, 61.8)

Notes. AUC refers to the mean areas under the drinking curves (calculated for each participant separately), given as M (SD). Larger AUCs (closer to 1) indicate more decelerated drinking. AUCs of 0.5 indicate linear drinking. AUCs closer to 0 indicate more accelerated drinking. Volume consumed at 50% time is calculated from cubic models of all participants' drinking trajectory data. Values given predict consumption at 50% time, with confidence intervals calculated by bootstrapping.

Table S5.
Study 2 - Baseline characteristics of participants by condition, and overall

	**Straight-sided (n = 36)**	**Outward-sloped (n = 36)**	**Overall (N = 72)**
**Gender (N)**			
Female	18	18	36
Male	18	18	36
**Highest Educational Qualification (%)**			
GCSE/O Level	2	1	3
AS/A Level	13	20	33
Undergraduate	9	11	20
Postgraduate	12	4	16
**Age (years)**	23.50 (19.75, 27)	20 (19, 22)	21.50 (19, 25.25)
**Thirst (1-10)**	5.94 (1.26)	5.42 (1.54)	5.68 (1.42)
**BMI (kg/m2)**	23.19 (3.06)	21.58 (2.86)	22.38 (3.05)
**Max oral capacity**	68.99 (20.09)	65.93 (19.28)	67.44 (19.60)

Notes. Thirst was rated from 1 (“Not at all”) to 10 (“Extremely”). Age is median (IQR); BMI and thirst are mean (SD). One participant omitted to complete the max oral capacity measure, in the straight-sided condition.

Table S6.

Study 2 - Unadjusted (univariate) and adjusted (multivariate) regression, predicting amount consumed (ml).

	**Unadjusted regression analyses**				**Adjusted regression analyses**		
Independent variable	*B*	95% CI (*B*)	*p*-value	R^2	*B*	95% CI (*B*)	*p*-value
(Intercept)	-	-	-	-	-49.45	-279.51 to 180.61	.675
**Glass shape**				.027			
Straight-sided	-55.70	-120.03 to 8.64	.089		-72.13	-132.56 to -11.69	.022
**Gender**				.042			
Male	64.92	1.09 to 128.76	.046		52.94	-19.15 to 125.02	.155
**Thirst (1-10)**	27.46	5.15 to 49.77	.017	.066	32.97	11.71 to 54.23	.003
**Drink enjoyment (1-10)**	18.82	-5.97 to 43.60	.135	.018	15.75	-7.07 to 38.57	.181
**Max oral capacity (ml)**	1.18	-0.51 to 2.87	.168	.013	0.513	-1.33 to 2.36	.588

Notes. Reference for Glass shape is Outward-sloped. Reference for Gender is Female. Thirst was rated from 1 (“Not at all”) to 10 (“Extremely”). Drink enjoyment was an average of “pleasant” and “tasty” ratings, which were rated from 1 (“Not at all”) to 10 (“Extremely”). Adjusted analyses: $F(5,65) = 3.928$, $p = .0036$, $R^2 = .173$.

Table S7.

Study 3 - Baseline demographics and characteristics of participants (N = 40)

**Gender (%)**	
Female	100
**Nationality (%)**	
Australian	62.5
Chinese	10.0
Bangladeshi	5.0
British	5.0
Other	17.5
**Highest Educational Qualification (%)**	
School Certificate (GCSE equivalent)	2.5
Higher School Certificate (AS/A Level equivalent)	60.0
Bachelors or Associate Degree	22.5
Postgraduate Degree	15.0
**Age (years)**	20 (19, 24.3)
**Thirst (1-10)**	5.48 (2.10)
**BMI (kg/m^2)**	23.06 (4.68)
**Max Oral Capacity (ml)**	52.46 (15.30)

Notes. Thirst was rated from 1 (“Not at all”) to 10 (“Extremely”). Age is median (IQR); BMI, thirst, and maximum oral capacity are mean (SD).

Table S8.
Study 3 - Linear mixed effects models predicting log(% MVC) upper, and log(%MVC) lower, from glass shape, adjusting for order effects.

	Upper Lip				***Lower Lip***			
	B	Exp(*B*)	95% CI Exp(*B*)	*p*-value	*B*	Exp(*B*)	95% CI Exp(*B*)	*p*-value
(Intercept)	2.632	13.91	10.61 to 18.22	< .0001***	2.487	12.02	9.11 to 15.86	< .0001***
**Glass shape**								
Straight-sided	0.086	1.089	1.033 to 1.148	.00168**	0.183	1.200	1.147 to 1.256	< .0001***
**Sequence**								
BABA	-0.097	0.908	0.625 to 1.318	.614	0.118	1.125	0.764 to 1.657	.555
**Drink number (1-4)**	0.0035	1.003	0.980 to 1.027	.773	-0.0269	0.973	0.954 to 0.993	.009**
**Sip number**								
2nd sip	-0.043	0.958	0.898 to 1.022	.198	-0.0199	0.980	0.928 to 1.036	.482
3rd sip	-0.065	0.937	0.878 to 1.000	.051	-0.0357	0.965	0.913 to 1.020	.207

Notes. % MVC is percentage of muscle activity used, as a proportion of maximum voluntary contraction (MVC). Participant ID was included as a random effect. Reference for Glass shape is Outward-sloped. Reference for Sequence is ABAB. Drink number is 1st, 2nd, 3rd, 4th. Reference for Sip number is 1st sip. Exp(*B*) = e^B.

Table S9.

Study 3 – Linear mixed effects models predicting log (sip size) from %MVC upper, and %MVC lower, adjusting for order effects, glass shape, thirst, and maximum oral capacity.

	Upper Lip				***Lower Lip***			
	B	Exp(*B*)	95% CI Exp(*B*)	*p*-value	*B*	Exp(*B*)	95% CI Exp(*B*)	*p*-value
(Intercept)	2.652	14.18	7.60 to 26.43	< .0001***	2.686	14.68	7.80 to 27.61	< .0001***
**%MVC**	-0.0019	0.998	0.994 to 1.002	.337	-0.0036	0.996	0.991 to 1.003	.251
**Glass shape**								
Straight-sided	-0.191	0.826	0.787 to 0.867	< .0001***	-0.182	0.834	0.792 to 0.878	< .0001***
**Sequence**								
BABA	-0.061	0.941	0.720 to 1.230	.669	-0.055	0.947	0.725 to 1.237	.701
**Drink number (1-4)**	-0.034	0.967	0.946 to 0.988	.0022**	-0.036	0.965	0.944 to 0.986	.0013**
**Sip number**								
2nd sip	0.017	1.017	0.959 to 1.079	.580	0.017	1.017	0.959 to 1.078	.580
3rd sip	0.024	1.024	0.965 to 1.086	.434	0.024	1.024	0.966 to 1.086	.426
**Thirst (1-10)**	-0.022	0.979	0.918 to 1.043	.524	-0.025	0.975	0.915 to 1.040	.468
**Max oral capacity (ml)**	0.002	1.002	0.993 to 1.011	.676	0.002	1.002	0.993 to 1.011	.661

Notes. % MVC is percentage of muscle activity used, as a proportion of maximum voluntary contraction (MVC). Participant ID was included as a random effect. Reference for Glass shape is Outward-sloped. Reference for Sequence is ABAB. Drink number is 1st, 2nd, 3rd, 4th. Reference for Sip number is 1st sip. Thirst was rated from 1 (“Not at all”) to 10 (“Extremely”). Exp(*B*) = e^B.
